# PD-L1 expression correlated with p53 expression in oral squamous cell carcinoma

**DOI:** 10.1186/s40902-019-0239-8

**Published:** 2019-12-05

**Authors:** Itaru Tojyo, Yukari Shintani, Takashi Nakanishi, Kenjiro Okamoto, Yukihiro Hiraishi, Shigeyuki Fujita, Mayu Enaka, Fuyuki Sato, Yasuteru Muragaki

**Affiliations:** 1 Department of Oral and Maxillofacial Surgery, Wakayama Medical University, 811-1 Kimiidera, Wakayama, Wakayama 641-8509 Japan; 2 Department of Dentistry and Oral Surgery, Japanese Red Cross Wakayama Medical Center, 4-20 Komatsubara-dori, Wakayama, Wakayama 640-8558 Japan; 3 Department of Pathology, Wakayama Medical University, 811-1 Kimiidera, Wakayama, Wakayama 641-8509 Japan

**Keywords:** Oral squamous cell carcinoma (OSCC), Programmed death ligand 1 (PD-L1), Protein 53 (p53), Cytokeratin 17 (CK17), Immunohistochemistry, Disease-specific survival rate

## Abstract

**Background:**

Programmed cell death ligand 1 (PD-L1) is an immune checkpoint molecule that attenuates the immune response. PD-L1 contributes to failed antitumor immunity; thereby, blockade of PD-L1 with monoclonal antibody enhances the immune response. Recently, it was reported that PD-L1 was regulated by protein 53 (p53). Besides, cytokeratin 17 (CK17) is thought to be a diagnostic marker of oral squamous cell carcinoma (OSCC). Our aim was to evaluate the correlation between the immunohistochemical expression of PD-L1, p53 and CK17 with clinicopathological characteristics and disease-specific survival in patients with OSCC.

**Methods:**

A total of 48 patients with OSCC were included in this study. Immunohistochemical staining was performed to evaluate the correlation among the expressions of PD-L1, p53 and CK17, and furthermore the correlation among various clinicopathological factors, PD-L1, p53 and CK17.

**Results:**

The positive rate of p53, CK17, PD-L1 (tumor cells) and PD-L1 (tumor-infiltrating lymphocytes) was 63.2%, 91.7%, 48.9% and 57.1%. A statistically significant correlation between p53 expression and T stage and TNM stage (*p* = 0.049, *p* = 0.03, respectively) was observed. Also, a statistically significant correlation between p53 and PD-L1 (TCs) expression (*p* = 0.0009) was observed. Five-year disease-specific survival rate was not significantly correlated with gender, TNM stage, p53 expression, PD-L1 expression and CK17 expression.

**Conclusion:**

The expression of p53 and PD-L1 shows significantly positive correlation in oral squamous cell carcinoma in tumor cells. Also, a significant correlation between p53 expression and T stage and TNM stage was observed. No other significant correlation between PD-L1 staining or CK17 and clinical or pathologic characteristics was identified.

## Background

Programmed death ligand 1 (PD-L1) is a cell-surface protein that has been proved to be overexpressed on various cells including tumor cells, lymphocytes and other tissues in many human cancers [1]. PD-L1 promotes T-cell tolerance and escape host immunity. It is reported that increased PD-L1 and PD-1 expression is predictive of nodal metastasis and poor prognosis in oral squamous cell carcinoma [2, 3].

Protein 53 (p53) is one of the most commonly mutated genes in cancer [4]. It is critical in regulating cell division, apoptosis, senescence and DNA damage and repair. p53 is also important for modulating the immune response. When DNA is damaged, p53 gene transcription is increased and wild-type p53 protein is concentrated, which result in the arrest of cell cycle at the G1/S phase and apoptosis of cancer cells. When the p53 gene is mutated, the mutant p53 protein loses its cancer inhibition function and promotes the transformation of normal cells to malignant cells. Mutant p53 is present in almost all types of human malignant tumor. Also, mutant p53 is closely correlated with oral squamous cell carcinoma [5–7].

Cytokeratins (CKs), intermediate filament of the cytoskeletons, are candidates for diagnostic markers of OSCC, as they are overexpressed in OSCC compared to normal mucosa. The significant up-regulation and the strong overexpression of CK17 could exhibit its utility as a diagnostic marker of OSCC [8–10].

In this study, we aimed to evaluate the correlation between the immunohistochemical expression of PD-L1, p53 and CK17 with clinicopathological characteristics and disease-specific survival in patients with OSCC.

## Methods

The study comprised a total of 49 selected patients (29 males and 20 females) with oral squamous cell carcinomas (OSCCs) at the Department of Oral and Maxillofacial Surgery, Wakayama Medical University Hospital, between 1990 and 2010. Patients were randomly selected. This study followed the Declaration of Helsinki on medical protocol and ethics, and the regional ethical review board of Wakayama Medical University approved the study (Protocol Identification Number 2512).

The patients ranged in age from 49 to 91 years, with a mean age of 65.7 years. The primary malignant tumors were located on the lower gingiva in 18 cases, the tongue in 11 cases, the upper gingiva in 5 cases, the oral floor in 7 cases, the maxillary sinus in 3 cases, the hard palate in 2 cases, and the soft palate in 3 cases. Tumor staging was performed according to the specifications of the TNM classification of malignant tumors (UICC 1997). The mode of tumor invasion was assessed according to the classification by Yamamoto et al as follows: grade 1 = well-defined borderline; grade 2 = cords, less marked borderline; grade 3 = groups of cells, no distinct borderline; and grade 4 = diffuse invasion (4C = cordlike type; 4D = widespread type) [11].

### Immunohistochemical staining

The PD-L1, P53 and CK17 expression OSCC tissues were evaluated from serial deparaffinized sections. The OSCC biopsy specimens had been fixed with formalin from 24 to 48 h at room temperature and treated with routine processing as in a previous study [12, 13]. Four micrometer-thick sections of paraffin-embedded tissues were mounted on precoated slides and air-dried overnight at 58°C. The serial sections were prepared for staining and were incubated with primary antibodies for 12 h. Immunohistochemistry (IHC) was performed using a Discovery Auto-Stainer with automated protocols (Ventana Medical Systems, Inc.; Roche) as previously described [13, 14].

The following commercial antibodies were purchased: PD-L1 (1:1, rabbit monoclonal, 790-4905; Ventana Medical Systems, Inc.; Roche, USA), p53 (1:100, mouse monoclonal, M7001; Dako Denmark, Glostrup, Denmark) and CK17 (1:50, mouse monoclonal, M7046; Dako Denmark, Glostrup, Denmark).

### Evaluation of staining results

**Fig. 1:**
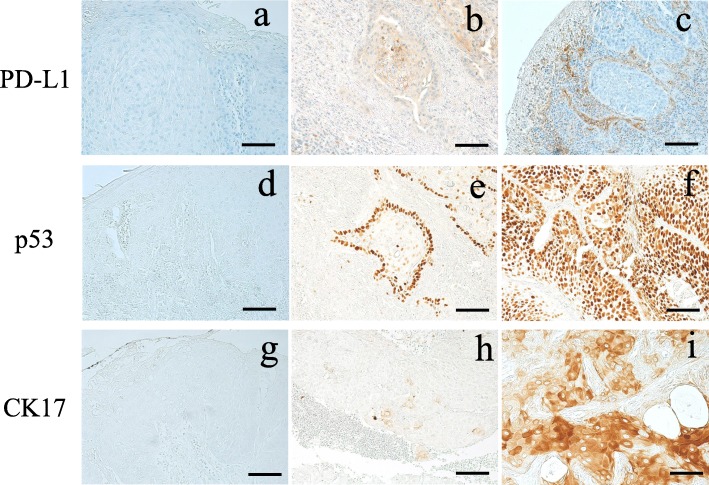
Representative images of PD-L1, p53 and CK17 expression by IHC. **a** PD-L1 expression negative. **b** PD-L1 expression of TCs moderate positive. **c** PD-L1 expression of TILs moderate positive. **d** p53 expression negative. **e** p53 expression moderate positive. **f** p53 expression strong positive. **g** CK17 expression negative. **h** CK17 expression weak positive. **i** CK17 expression strong positive. Scale bars: 100μm

The tumor biopsy sample was considered positive for PD-L1 expression in tumor cells if moderate-to-strong membrane staining was observed in ≥5% of tumor cells (TCs) based on previous larger studies (Fig.1a, b). Tumor-infiltrating lymphocytes (TILs) were considered to have positive PD-L1 expression if ≥1% of TILs exhibited moderate-to-strong membrane staining, as previously described (Fig. 1a, c) [7].

p53 expression was considered positive when ≥10% of the TC nuclei (Fig. 1d, e, f) [7].

The CK17-stained sections with <5 % reactive cells were considered to be negative, and those with more than 5 % reactive cells were defined as positive (Fig. 1g, h, i) The sections were divided into two groups as follows: over 60 % positive cells were defined as “strong” cases; less than 60 % positive cells were defined as “weak” cases [8].

The Chi-square (*χ*2) test and Fisher’s exact test were used to analyze differences in categorical variables (including gender, age, TNM stage, differentiation, mode of invasion) between positive PD-L1/P53/CK17 expression groups and negative PD-L1/P53/CK17 expression groups. In addition, univariate survival analysis and survival curves were conducted by Kaplan–Meier method and the log-rank test was routinely used to test for significant differences.

## Results

The differentiation degree was well differentiated in 35 cases, moderately differentiated in 11 and poorly differentiated in three cases. The mode of invasion was grade 1 in 3 cases, grade 2 in 9, grade 3 in 17, grade 4C in 16 and grade 4D in 4 cases. Positive CK17, p53, PD-L1 (TCs) and PD-L1 (TILs) staining was present in 91.7%, 63.2%, 48.9% and 57.1% of all cases, respectively.

Table 1 shows the clinical and pathologic characteristics associated with PD-L1, p53 and CK17 expression. With respect to tumor stage, a statistically significant correlation between p53 expression and T stage and TNM stage (*p* = 0.049, *p* = 0.03, respectively) was observed. Also, a statistically significant correlation between p53 and PD-L1 (TCs) expression (*p* = 0.0009) was observed. No other significant correlation between PD-L1 staining or CK17 and clinical or pathologic characteristics was identified (Table 1).

**Table 1:** Characteristics of PD-L1/p53/CK17 expression in patients with oral squamous cell carcinoma

Characteristics			PD-L1 expression in TCs			p53 expression			CK17 expression		
		Cases	Positive ( ≥5%)	Negative (<5%)	*P* value	Positive ( ≥10%)	Negative (<10%)	*P* value	Strong ( ≥60%)	Weak (<60%)	*P* value
Gender	Male	29	14	15	1.00	20	9	0.37	4	25	0.27
	Female	20	10	10		11	9		6	14	
Age	<65 years	22	12	13	1.00	15	10	0.76	5	20	1.00
	>65 years	27	12	12		16	8		5	19	
T stage	T1–T3	34	15	19		18	16		5	29	0.12
	T4	14	8	6	0.52	12	2	0.049^*^	5	9	
	Unknown	1	1	0		1	0		0	1	
N stage	N (+)	20	10	10		13	7		4	16	
	N (-)	28	13	15	1.00	17	11	1.00	6	22	1.00
	Unknown	1	1			1				1	
M stage	M0	48	22	25		29	18		0	1	
	M1	0	1	0	0.47	1	0	1.00	10	37	1.00
	Unknown	1	1	0		1	0		0	1	
TNM stage	I–III	30	13	17		15	15		4	26	
	IV	18	10	8	0.55	15	3	0.03*	6	12	0.14
	Unknown	1	1	0		1	0		0	1	
Differentiation	Well	35	19	16		20	15		8	27	
	Moderate	11	4	7	0.54	8	3	0.31	1	10	0.47
	Poor	3	1	2		3	0		1	2	
Mode of invasion	Grade 1	3	2	1		2	1		2	1	
	Grade 2	9	4	5		3	6		1	8	
	Grade 3	17	9	8	0.96	11	6	0.21	3	14	0.37
	Grade 4C	16	7	9		11	5		3	13	
	Grade 4D	4	2	2		4	0		1	3	
PD-L1 expression in TCs	Positive	24				21	10	0.0009*	17	22	0.17
	Negative	25				3	15		7	3	
PD-L1 expression in TILs	Positive	28	21	7	< .0001*	21	7	0.07	20	19	0.15
	Negative	21	3	18		11	10		8	2	
p53 expression	Positive	31	21	10	0.0009*				22	17	0.06
	Negative	18	3	15					9	1	
CK17 expression	Weak	39	17	22	0.17	22	17	0.06			
	Strong	10	7	3		9	1				

*P* value : χ^2^ test and Fisher's exact test were used. *P<0.05 was defined as significantly different in statistical analysis

Unknown includes those cases with Tx, Nx, Mx and TNM status and they were excluded from statistical analysis

**Fig. 2:**
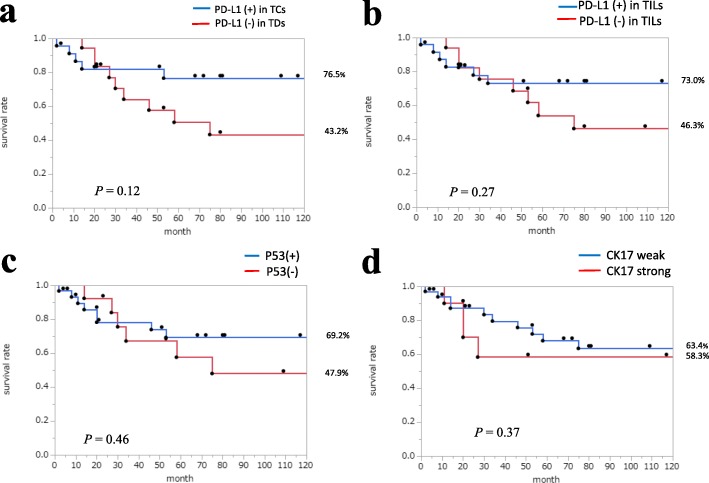
Kaplan–Meier curve and long-rank test for 5-year disease-specific survival rate. **a** PD-L1 expression in TCs. **b** PD-L1 expression in TILs. **c** p53 expression. **d** CK17 expression

However, the 5-year disease-specific survival rate by Kaplan–Meier method of the cases with PD-L1 (no expression) or CK17 (strong expression) tended to be slightly low, but not significant (Fig. 2a, b, d). Five-year disease-specific survival rate was not significantly correlated with p53 expression (Fig. 2c). The survival rate was not significantly correlated even with PD-L1 (+) and p53 (+).

Hazard ratio indicated that the 5-year disease-specific survival rate was not significantly correlated with gender, TNM stage, p53 expression, PD-L1 expression, and CK17 expression (Table 2).

**Table 2:** Prognostic factors for disease-specific survival of the patients with oral squamous cell carcinoma according to multivariate analysis

Characteristics	Group	Hazard ratio (95%CI)	*P* value	
Gender	Female/male	0.389 (0.094-1.610)	0.193	
T stage	T1–T3/T4	0.818 (0.077-8.673)	0.867	
N stage	N(-)/N (+)	0.860 (0.262-2.829)	0.805	
TNM stage	I–III/IV	1.259 (0.088-17.941)	0.864	
PD-L1 expression in TCs	Positive/negative	0.412 (0.111-1.530)	0.185	
p53 expression	Positive/negative	0.655 (0.176-2.440)	0.528	
CK17 expression	Strong/weak	3.418 (0.806-14.49)	0.095	

*P<0.05 was defined as significant difference in statistical analysis

## Discussion

In this study, it is shown that the expression of PD-L1 is correlated with the expression of p53 in oral squamous cell carcinoma.

PD-L1 overexpression is recognized in many human cancers, promoting T-cell tolerance and escape host immunity. Early clinical trials using monoclonal antibodies that block the PD1/PDL1 interaction have shown promise in some patients with advanced cancer. OSCC patients with high PD-L1 expression had poor clinical outcome and might require PD-L1-targeted immunotherapy to improve their prognosis. Mutant p53 is present in almost all types of human tumor and is closely correlated with the development of OSCC. Mutated p53 loses its ability to suppress the function of oncogenes. Furthermore, mutant p53 may function as an oncogene to stimulate cell division and promote the growth of tumor cells [6].. Although whether p53 is involved in tumor immune evasion has been poorly understood, Cortez reported that PD-L1 is regulated by p53 via micro RNA (miR-34a) using a series of experiments involving lung cancer cell lines [15].

Regarding tumor cells, the expression of PD-L1 and p53 is positively correlated, because wild-type p53 is rapidly degraded (~0.5h); however, as the resolution time of variant p53 protein is delayed (›2h) and the protein is accumulated in the nucleus, the variant p53 protein is identified as overexpression [16, 17]. Although wild-type p53 inhibits the expression of PD-L1 directly, when variant p53 which has lost a function is accumulated, PD-L1 is overexpressed. Thus, it is thought that the expressions of p53 and PD-L1 show positive correlation in oral squamous cell carcinoma in this study.

Furthermore, based on the results of Cancer Genome Atlas exome data analysis, there is a link between P53 status and mutation burden in tumors [18]. That is to say that the evaluation of P53 status could be used as a surrogate biomarker for mutation burden [19]. At the same time, although many different factors modulate the clinical response to an immune checkpoint inhibitor, the strong relationship between the tumor mutational burden and the activity of anti-PD-1 therapies across multiple cancers has been highlighted and the association of p53 and PD-L1 also suggested.

## Conclusion

In this study, the expression of p53 and PD-L1 shows a positive correlation in oral squamous cell carcinoma in tumor cells for the first time. No other significant correlation between PD-L1 staining or CK17 and clinical or pathologic characteristics was identified.

## Data Availability

Please contact the author for data requests.

## Abbreviations

CK17: Cytokeratin 17
IHC: Immunohistochemistry
OSCC: Oral squamous cell carcinoma
p53: Protein 53
PD-L1: Programmed cell death ligand 1
TCs: Tumor cells
TILs: Tumor-infiltrating lymphocytes
